# Retrograde intrarenal surgery for impacted upper ureteral stone in a patient with advanced lumbar scoliosis and lower-extremity development defect: a case report

**DOI:** 10.1186/s13256-022-03411-w

**Published:** 2022-05-26

**Authors:** Yavuz Güler

**Affiliations:** Private Safa Hospital, Rumeli Universty, İstanbul, Turkey

**Keywords:** Ureteral stone, Impacted stone, Sepsis, Scoliosis, Retrograde intrarenal surgery, Ureterorenoscopy

## Abstract

**Background:**

Today, retrograde intrarenal surgery is the most preferred and very successful treatment method for upper ureteral stones that do not spontaneously pass and/or do not benefit from extracorporeal wave lithotripsy. However, perioperative complications are more common in retrograde intrarenal surgery if the stone in the ureter is impacted. Moreover, urosepsis and renal dysfunction are detected more frequently in patients with impacted stones. Impacted stones, which are a risky stone group even in patients with normal vertebral anatomy, are a more challenging situation in patients with advanced vertebral scoliosis. It is difficult to achieve an operating position in these patients. In addition, the ureteral tracing is altered, curved, and tortuous, making it more difficult for the endoscope to advance through the ureter.

**Case presentation:**

In this case report, we present a 23-year-old Caucasian male patient with right concavity and severe scoliosis, lower-extremity developmental disorder, and urosepsis. To treat the urosepsis picture, first percutaneous nephrostomy drainage was provided and the urine was sterilized with appropriate antibiotics according to the culture/antibiogram. Then, we performed ureterolithotripsy with a flexureterorenoscope. Finally, we see that flexible ureterorenoscopic lithotripsy to the upper ureteral stone with impacted stones, which is a very challenging operation even in patients with normal vertebrae, could be successfully performed in our patient with advanced scoliosis deformity.

**Conclusion:**

High stone-free and low complication rates can be obtained with flexible ureterorenoscopic retrograde intrarenal surgery in medium-sized impacted upper ureteral stones in patients with advanced scoliosis.

## Background

A normal vertebral column does not have curvature to the right or left in the sagittal plane. Situations with curvature to the right or left are called scoliosis. When the Cobb angle is > 90°, it is referred to as advanced scoliosis [1]. Patients with spinal deformities are reported to be predisposed to urolithiasis due to immobility, bacteriuria, and urinary stasis (0.24% for people with normal spines, 1.4–4.03% for those with spinal deformities) [2].

Impacted ureter stones are the most difficult stone group even in patients with normal vertebral structure [3]. These stones cause inflammation, intraluminal polyposis, and fibrosis of the ureter wall linked to chronic irritation of the ureter wall when the stones remain in the ureter lumen for long durations. The lack of a gap between the ureter lumen and the stone with impacted stones reduces the fragmentation effect of ESWL energy. Even if the stone does fragment, spontaneous stone discharge does not occur as the broken fragments adhere to the ureter wall. In RIRS, mainly problems with endovision are experienced due to microhemorrhage, larger volume of impacted stones, and intraluminal polyps. Additionally, delayed opening of the passage proximal of the stone may disrupt the view of fragmented mini stones and dust particles, making work more difficult. For this reason, RIRS for impacted upper ureter stones varies according to the experience of the surgeon, and unsuccessful operation, incomplete stone fragmentation, ureter perforation, and sepsis are observed more frequently. As a result, PCNL (antegrade ureterolithotripsy), open ureterolithotomy, and laparoscopic ureterolithotomy surgeries may be required for these patients [4].

In patients with vertebral scoliosis, location and shape deformities of intraabdominal organs may be observed, especially on the side of the scoliosis convexity. For this reason, there are natural difficulties in upper ureter stone treatment for these patients. In ESWL, apart from positioning the patient, an inability to site the energy head on the skin, and immobilization of the patient, the main difficulty is the lack of spontaneous passing of the broken fragments. For PCNL, the narrow interval between the costal margin and CIAS, neighboring organs within the percutaneous access route, and increased risk of postoperative sepsis due to contamination of these patients with chronic bacterial agents are disadvantages [5]. For RIRS, the main disadvantages are difficulties positioning the patient for dorsal lithotomy, access difficulties linked to ureter tortuosity and kinking due to the ureter passing below the immediate ventral of the vertebral bone structure, and difficulties with spontaneous passing of stone fragments due to immobilization of the patient and sepsis.

In our case, we present RIRS performed for a patient with advanced scoliosis and convexity toward the upper ureter containing the stone, attending with pyonephrosis and urosepsis due to impacted upper ureter stone obstruction.

## Case presentation

The 23-year-old Caucasian male patient, with advanced lumbar scoliosis of J-shaped convexity toward the left (toward the ureter with stone) and paraplegia, presented with high fever and disordered general status. Inspection found the patient was pale and tired, and physical examination found left costa-vertebral and left abdominal sensitivity. Family history was unremarkable.

At the first visit, height and weight of the patient were 165 cm and 74 kg, respectively. Blood pressure was 117/84 mmHg, heart rate was 95 beats/minute, and body temperature was 38.3 °C. The patient had no history of smoking or drinking. On physical examination, he had a lower-extremity developmental and neurological defect that prevented him from walking. He had used ciprofloxacin 500 mg twice a day before presenting to us.

The patient first had routine biochemical blood and urine analyses, including WBC, CRP, creatinine, urine analysis, and urine culture/antibiogram, and urinary USG and non-contrast full abdominal tomography were requested for imaging. CRP and WBC values were high (320 g/L, 17 × 10^3^, respectively). Urine analysis revealed microscopic hematuria with red blood cells (RBC) 10–15/high-power field (hpf) and microscopic leukocyturia with white blood cells (WBC) 20–25/hpf.

**Fig. 1:**
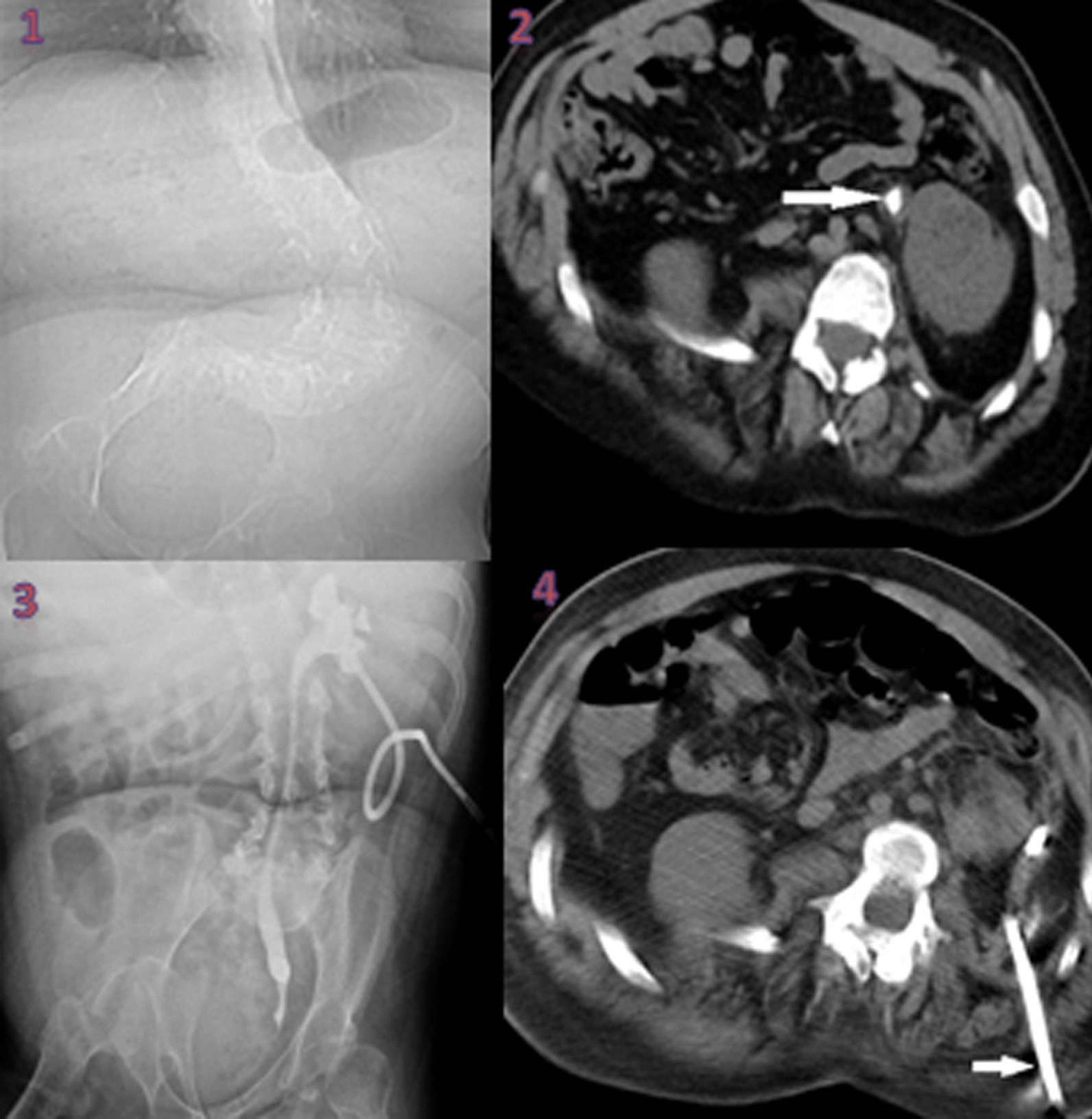
**1** Severe scoliosis with right-facing opening. **2** Impacted stone at the upper of the left ureter (indicated by arrow). **3** Antegrade ureterorenography. **4** Nephrostomy tract (indicated by arrow)

Blood creatinine, liver functions, and other biochemical tests showed no problem. Urinary system ultrasonography and noncontrast full abdominal tomography identified a 15 × 8 mm^2^ stone in the left upper ureter and linked grade 2 hydronephrosis. Firstly, we wished to insert a DJ stent under general anesthesia with the aim of providing urinary drainage for the left kidney. However, the guide wire could not progress past the lower ureter (probably due to tortuosity), so the patient was turned to prone position in the same session (the interval from costal margin to CIAS was very narrow), and the posterior lower calyx of the kidney was entered with 18G needle through this narrow interval accompanied by ultrasound and a 14F percutaneous nephrostomy catheter was inserted (Fig. 1). A sample of material coming from the nephrostomy was sent for culture-antibiogram tests. From these, *Escherichia coli* proliferated. As a result of the culture antibiogram, the multidrug-resistant bacterial agent was sensitive to the carbapenem group, and 1 g meropenem treatment 3 times a day was given in the hospital for a total of 10 days. After appropriate antibiotic treatment, nephrostomy and bladder urine were identified to be free of biological agents. Then, after receiving patient consent, RIRS was planned with a flexible ureterorenoscope. The patient was administered general anesthesia and then placed in dorsal lithotomy position. The bladder was reached with a 7.5 rigid ureterorenoscope, and a guide wire was sent into the right ureter. The lower tip of the ureter was dilated with the rigid ureterorenoscope. As the access sheath could not progress above the guide wire, the kidney was reached directly with the flexible ureterorenoscope. The stone was at the upper tip of the ureter and was fragmented with Ho:YAG laser (1 J power, 10 Hz frequency). At the end of the procedure, a DJ stent was inserted. The nephrostomy catheter was removed. The procedure was ended. There were no complications in the peri- or postoperative period. He stayed in the hospital for two nights and one day postoperatively. He was discharged on the second postoperative day. The patient had the DJ stent removed at the end of the fourth week. No problem was observed in the left kidney at 1, 3, and 6 months after DJ stent removal.

## Discussion

Stone impaction on the ureteral wall is a negative predictive factor that decreases the success of RIRS and increases complication rates in upper ureteral stones. Stone remaining in the ureteral lumen for a long time causes constant irritation in the ureteral wall. As a result of this irritation, inflammation, edema, polyps, and fibrosis occur in the ureteral wall. All these processes and easy mucosal hemorrhage as a result of irritation adversely affect endovision in RIRS. At the same time, large stone volume and poor endovision quality of buried stones may disorient the operator. Sometimes, because the stone cannot be completely broken (partial lithotripsy), the proximal pathway cannot be opened and renal drainage cannot be provided. As auxiliary therapy, percutaneous nephrostomy drainage may be needed. Major complications such as perforation of the ureteral wall and avulsion of the ureter can be seen during RIRS. These problems encountered in patients with normal vertebral curvature may be more advanced in patients with abnormalities in the vertebral curvature such as scoliosis. In the literature, it has been reported that stone-free rates of 75% [6] and 87.5% [7] were obtained without major complications in patients with medium-sized kidney stones with spinal deformity with RIRS. We know that percutaneous nephrolithotomy operations with an antegrade approach have been successfully performed in patients with upper ureteral stones [8, 9] with spinal deformity. However, we could not find any study on RIRS in patients with advanced scoliosis with impacted upper ureteral stones.

Treatment of impacted upper ureter stones is very challenging even in patients with normal spinal anatomy [3, 4]. As in patients with normal spinal anatomy, the main treatment choices for patients with impacted upper ureter stones and vertebral deformities are ESWL, RIRS, PCNL, and open/laparoscopic ureterolithotomy [3, 4]. In patients with advanced lumbar scoliosis where the convexity is toward the side with the kidney stone, narrowing the interval between the costal margin and CIAS, it appears difficult to obtain stone-free status due to reasons such as positioning in ESWL, inability to achieve full contact between the ESWL energy head and the patient, and difficulty with spontaneous passing of stone fragments due to immobilization of the patient even if ESWL can be performed. ESWL is reported to have low stone clearance rates (44–73%) in patients with vertebral deformities [10].

For stones larger than 2 cm, and/or complex partial or staghorn stones, PCNL is known to be the gold-standard treatment [11]. For stones at the upper end of the ureter, PCNL is indicated for patients where methods such as ESWL and RIRS will not be successful. PCNL or M-PCNL is a very effective/successful method to obtain stone-free status, but major peri- and postoperative complications should be remembered. Additionally, in patients with spinal deformity due to advanced scoliosis, risks further increase due to the narrow interval for percutaneous access in standard prone position, increased organ injury risk, and cardiopulmonary and anesthetic risks linked to position. Classic prone, lateral decubitus, and supine PCNL positions may be considered for this surgery according to the degree of deformity of the patient; if possible, fluoroscopic access should be performed accompanied by US [12]. Laparoscopic or open ureterolithotomy surgeries may be chosen if it is understood that other methods will not or cannot be successful for stones at the upper end of the ureter [11]. However, like PCNL, morbidity is higher in laparoscopic and open ureterolithotomy surgeries compared with RIRS and ESWL, and these surgical methods have steep and long learning curves, requiring surgical experience.

Thin flexible endoscopes with 270° flexion capability and laser fiber innovations today allow endoscopic ureterolithotomy surgeries for upper urinary tract stones of nearly all sizes and numbers to be performed by entering the native ureter orifice of the patient to reach the upper ureter and renal collecting system. In fact, even for large stones where PCNL is contraindicated, it may be possible to remove kidney stones with several sessions of RIRS.

Before RIRS, it is not routine to use preoperative DJ stent. It is necessary to use preoperative DJ stent due to ureter stenosis, pyonephrosis, and sepsis. In our patient, on first attendance, the clinical status was severe left renal colic pain, high CRP and WBC values, and fever episodes. For this reason, we identified the patient’s clinical diagnosis as pyonephrosis and urosepsis developing secondary to stone obstruction. Then, we performed emergency renal drainage. Due to tortuosity of the lower ureter, a 0.0038-inch guide wire could not pass proximal of the lower ureter, so the patient was placed in prone position in the same session and a 14F percutaneous nephrostomy catheter was inserted accompanied by US. After the patient’s urine and blood culture/antibiogram tests were cleared of bacterial agents, we performed RIRS.

When working with RIRS in the upper urinary tract, it is recommended that working at low pressure by lowering intrarenal hydrostatic pressure makes it easier to prevent major complications such as sepsis and renal capsular hematoma and ureteral access sheaths (UAS) should be used allowing reentry into the ureter. The risk of UTI and sepsis are reported to increase as the number of stones and dimensions increase in RIRS and the surgical duration lengthens. For this reason, UAS gains more importance to prevent complications in patients with large kidney stones and lengthened surgical duration. however, even in patients with normal vertebral anatomy, UAS may not advance due to reasons such as ureter tortuosity and ureter stenosis. In fact, in our patient, we could not insert UAS due to stenosis or tortuosity of the lower end of the ureter. We think we did not observe peri- and postoperative sepsis and/or renal capsular hematoma and extravasation due to reasons such as the lack of bacterial agent proliferation in urine before surgery, the surgery not lasting longer than 1 hour, and open drainage of the percutaneous nephrostomy catheter during the surgery. In a study of pediatric ureter stone patients with and without spinal deformity, Colangelo *et al.* [13] identified serious differences in stone-free rate (SFR) and complication rates for patients with spinal deformity in favor of patients without spinal deformity (success and complications, 61% versus 35.7% and 6.1% versus 40%, respectively). In our case, we did not encounter any complications.

This case report is the first to present RIRS performed for impacted upper ureter stone causing urosepsis in a patient with advanced scoliosis and paraplegia.

## Conclusion

Even for impacted upper ureter stones of moderate size in advanced scoliosis cases with convexity toward the stone side, high stone-free rates and low complication rates may be obtained with flexible ureterorenoscopic RIRS.

## Availability of data

Not applicable.

## Abbreviations

RIRS: Retrograde intrarenal surgery
ESWL: Extracorporeal wave lithotripsy
PCNL: Percutaneous nephrolithotomy
CIAS: Crista iliaca anterior superior
WBC: White blood cell
CRP: C-reactive protein
USG: Ultrasonography
DJ: Double J
M-PCNL: Mini-percutaneous nephrolithotomy
UAS: Ureteral access sheath
UTI: Urinary tract infection
SFR: Stone-free ratio
